# PMAIP1 regulates the progression of follicular thyroid carcinoma through the Wnt3/FOSL1 pathway

**DOI:** 10.3389/fonc.2025.1502391

**Published:** 2025-01-29

**Authors:** Haobo Wang, Fangjian Shang, Yifang Wang, Bo Pang, Longfei Kang, Chuanmin Zhou, Dongyun Li, Zhongxin Li, Xia Jiang, Bo Liu, Zengren Zhao

**Affiliations:** ^1^ Department of General Surgery, The First Hospital of Hebei Medical University, Shijiazhuang, China; ^2^ Gastrointestinal Disease Diagnosis and Treatment Center, Hebei Key Laboratory of Colorectal Cancer Precision Diagnosis and Treatment, The First Hospital of Hebei Medical University, Shijiazhuang, China

**Keywords:** follicular thyroid carcinoma, PMAIP1, Wnt3, FOSL1, progression

## Abstract

**Introduction:**

In thyroid carcinoma (TC), follicular thyroid carcinoma (FTC) represents the second most prevalent pathological type following papillary thyroid carcinoma. Notably, FTC exhibits a more aggressive clinical course and a higher propensity for distant metastasis. However, the underlying mechanisms governing the progression of FTC remain poorly understood. PMAIP1 is a gene implicated in various cancers and biological processes. Investigating the role and mechanism of PMAIP1 in FTC is crucial for enhancing our understanding of FTC and informing clinical treatment strategies.

**Methods:**

This study examined the expression level of PMAIP1 in FTC through comprehensive analysis of databases, tumor tissues, and cell lines. Following the establishment of a stably transfected plasmid in cell lines, a series of functional assays and subcutaneous xenograft experiment were conducted to investigate the role of PMAIP1 in FTC. Additionally, transcriptome sequencing was employed to identify potential signaling pathways associated with PMAIP1. Mechanistic studies involved a series of rescue experiments to elucidate the regulatory mechanisms of PMAIP1 in FTC.

**Results:**

PMAIP1 was found to be highly expressed in FTC, and its knockdown significantly inhibited the proliferation and metastasis of FTC cells both *in vivo* and *in vitro*. The results of transcriptome sequencing analysis indicated that PMAIP1 may influence the progression of FTC via the Wnt signaling pathway. Subsequent investigations revealed a direct correlation between PMAIP1 expression levels and those of Wnt3 and FOSL1 in FTC. A series of rescue experiments further substantiated the regulatory role of PMAIP1 on Wnt3/FOSL1 in FTC.

**Discussion:**

In conclusion, our research demonstrated that PMAIP1 emerges as a novel pro-cancer factor in FTC, and its knockdown significantly inhibited the proliferation and metastasis of FTC both *in vivo* and *in vitro*. Mechanistically, PMAIP1 regulated FOSL1 by modulating the Wnt signaling pathway, thereby promoting FTC progression. Targeting PMAIP1 may present a promising therapeutic strategy for FTC.

## 1 Introduction

Thyroid cancer (TC) is the most prevalent endocrine malignancy, accounting for 586,000 cases globally and ranking ninth in incidence in 2020 (1, 2). The increased utilization of diagnostic imaging and surveillance has revealed a clear trend of rejuvenation in TC, making it a significant type of cancer that warrants attention (3). Among differentiated thyroid cancers, follicular thyroid carcinoma (FTC) has an incidence rate of approximately 10% to 15%, second only to papillary thyroid carcinoma (PTC), but it is notably more invasive (4). FTC is considered a high-risk cancer due to its propensity for hematogenous metastasis to distant sites, particularly the lungs and bones (5). Despite significant advancements in the diagnosis and treatment of FTC, the underlying mechanisms of FTC remain largely obscure. Consequently, investigating potential biomarkers and elucidating their molecular mechanisms in FTC is of paramount importance to address this research gap. Concurrently, such investigations may facilitate the development of novel therapeutic targets for FTC, thereby expanding the repertoire of clinical treatment options.

PMAIP1 (Phorbol-12-myristate-13-acetate-induced protein 1), also known as NOXA or APR, located on chromosome 18q21.32, is a member of the BCL-2 protein family (6). Research indicated that insulin activates the AKT signaling pathway, which subsequently inhibits the RNA translation of NOXA/PMAIP1, thereby promoting the survival of human pluripotent stem cells (7). NOXA/PMAIP1 has the potential as a predictive marker for response and survival in patients undergoing CAR T-cell transfusion. Targeting NOXA/PMAIP1 may enhance the therapeutic efficacy of CAR T cells. However, the functional significance of PMAIP1 in FTC remains unexplored (8). Furthermore, azacitidine was observed to upregulate the expression of PMAIP1, thereby enhancing the sensitivity of preclinical models of acute myeloid leukemia to venetoclax. This finding provided evidence for novel therapeutic strategies aimed at overcoming resistance to current acute myeloid leukemia treatments (9). These studies indicated that PMAIP1 plays a significant role in the biological progression of various diseases and may serve as a potential therapeutic target. Additionally, PMAIP1 was implicated in the pathogenesis and progression of multiple cancers. Research indicated that NOXA/PMAIP1 serves as a marker for an aggressive subtype of pancreatic ductal adenocarcinoma, with NOXA/PMAIP1 expression inducing synthetic lethality upon RUNX1 inhibition in pancreatic cancer (10). The co-expression of PUMA and NOXA/PMAIP1 proteins in benign epithelial cells has been predictive of recurrence following radical prostatectomy (11). Additionally, a study involving 160 patients with colorectal cancer and adjacent tissues demonstrated that NOXA/PMAIP1 was overexpressed in colorectal cancer tumors, even at early stages (12). However, the functional significance of PMAIP1 in FTC remains unexplored.

In this study, we conducted a comprehensive analysis utilizing the databases, supplemented by validation with clinical tumor samples and cell lines, and identified elevated expression levels of PMAIP1 in FTC. Consequently, we postulated that PMAIP1 contributes to the progression of FTC. To substantiate this hypothesis, we examined the impact of PMAIP1 on FTC proliferation and metastasis through both *in vivo* and *in vitro* experiments. Additionally, we employed transcriptome sequencing to elucidate the potential pathway through which PMAIP1 may exert its effects in FTC, and subsequently verified the association between PMAIP1 and the pathway by rescue experiments. In this study, we examined the expression levels, functional roles, molecular mechanisms, and clinical significance of PMAIP1 in FTC.

## 2 Materials and methods

### 2.1 Clinical samples

Pairs of FTC and adjacent non-tumorous tissue specimens (n=8) were procured from the Department of General Surgery at The First Hospital of Hebei Medical University. The study received ethical approval from the Ethics Committee of The First Hospital of Hebei Medical University (NO: 20230203), and informed consent was obtained from all patients for the collection of tissue samples.

### 2.2 Bioinformatics analysis

RNA-sequencing expression profiles and corresponding clinical data of FTC were obtained from The Cancer Genome Atlas (TCGA) dataset (https://portal.gdc.cancer.gov/). Normal control datasets were sourced from the Genotype-Tissue Expression (GTEx) data portal (https://www.gtexportal.org/home/datasets). Statistical analyses were conducted using R software (Version 3.5.0). The two-gene correlation map was generated using the ggstatsplot package in R, employing Spearman’s correlation analysis to assess the relationship between quantitative variables that do not follow a normal distribution. *P*<0.05 was considered statistically significant.

### 2.3 Cell lines

The FTC cell lines (FTC133 and FTC238) and the non-tumoral thyroid cell line (NTHY-ORI3-1) were used in this study. The human FTC orthotopic cell lines FTC133 (1×10^6^) and the non-tumoral thyroid cell line NTHY-ORI3-1 (1×10^6^) were procured from Procell Life Science&Technology Co, Ltd (Cat NO.: CL-0644; CL-0817). The human FTC lung metastasis cell line FTC238 was procured from YUCHI Biology Co, Ltd (Cat NO.: SC-1458). FTC133 and NTHY-ORI3-1 were maintained in RPMI-1640 medium (Thermo, Gibco, UK) and FTC238 was maintained in DMEM F-12 medium (Thermo, Gibco, UK), all supplemented with 10% fetal bovine serum (Thermo, Gibco, UK) and 1% penicillin-streptomycin (Thermo, Gibco, UK). All cell lines were incubated at 37°C with 5% CO_2_.

### 2.4 RNA extraction and quantitative real−time polymerase chain reaction

Total RNA was extracted utilizing the RNA EASY reagent (R701-01, Vazyme, China), followed by reverse transcription with the Prime Script RT Reagent Kit (RR047A, TaKaRa, Japan). The Quantitative real‑time polymerase chain reaction (qRT‑ PCR) system (LightCycler 480II, USA) was prepared using the ChamQ Universal SYBR qPCR Master Mix (Q711, Vazyme, China), cDNA, and specific primers. PCR amplification reactions were conducted in triplicate for each cDNA sample, with β-actin serving as the internal reference gene. All gene-specific primers were designed using NCBI (https://www.ncbi.nlm.nih.gov/) resources and synthesized by Sangon Biotech Co., Ltd (Shanghai, China) (**Table 1**). Relative gene expression levels were calculated using the 2^−ΔΔCt^ method.

**Table 1:** Primer sequences used in this study.

Gene name	Primer sequences	Length (bp)
PMAIP1	F: AGGAACAAGTGCAAGTAGCTG	153
	R: GGAGTCCCCTCATGCAAGTT	
Wnt3	F: TGACTCGCATCATAAGGGGC	181
	R: GTGGTCCAGGATAGTCGTGC	
FOSL1	F: GTGCCAAGCATCAACACCAT	126
	R: CCAGTTTGTCAGTCTCCTGTTC	
β-actin	F: ACTTAGTTGCGTTACACCCTT	155
	R: GTCACCTTCACCGTTCCA	

### 2.5 Establishment of knockdown and overexpression cell lines

Knockdown plasmid of PMAIP1 (sh-PMAIP1: 5’-GTGCTACTCAACTCAGGAGAT -3’), sh-negative control (sh-NC), over-expression plasmid of PMAIP1 (NM_001382618.1), Vector, knockdown plasmid of Wnt3 (sh-Wnt3: 5’-GCGCTTCTGCCGCAATTACAT-3’), siRNA of FOSL1 (siFOSL1:5’-CCUCAGCUCAUCGCAAGAGUA-3’) and siNC were ordered form Guangzhou Fulengen Co, Ltd. 5μg plasmids (or 5μmol siRNA) were transfected into FTC133 and FTC238 by using Lipofectamine 3000 (Invitrogen, USA) according to the manufacturer’s instructions. The cells were then selected using 1 µg/mL puromycin (Solarbio, China).

### 2.6 Western blot analysis

Protein was extracted from the cells using RIPA lysis buffer (PMSF: RIPA = 1:100), and the protein concentration was semi-quantified using a BCA protein assay kit. Equal amounts of protein were separated by 12% SDS-PAGE (BIO-RAD kit) and subsequently transferred to a PVDF membrane. The PVDF membrane was blocked with 5% BSA in TBST at room temperature for 2 hours. Following three washes with TBST, the membrane was incubated with primary antibody at 4°C overnight. Afterward, it was washed three times with TBST and then incubated with a secondary antibody at room temperature for one hour. Finally, the protein bands were visualized and analyzed. This experiment was repeated three times.

### 2.7 Cell counting kit−8 assay

Cells were digested and quantified during the logarithmic growth phase, subsequently seeded in 96-well plates at a density of 1x10^3^ cells per well, and incubated at 37°C with 5% CO_2_. Each experimental group included four replicates. Upon cell adherence, 10µL of cell counting kit‑8 (CCK‑8) reagent (Abbkine, China) was added to each well at 0h, 24h, 48h, and 72h, followed by incubation at 37°C. After a 2h incubation period, the absorbance of the 96-well plates was measured at 450 nm using a microplate reader.

### 2.8 Colony formation assay

Cells were digested and counted during the logarithmic growth phase, and 2mL of the medium was added to a 6-well plate, with the cell suspension seeded at a density of 1,000 cells per well. Cells were cultured in a 5% CO_2_ incubator at 37°C for a duration of 10-14 days, with the medium being replaced every 3 to 4 days. The experiment was terminated upon the observation of significant clonal cell groups. Following the removal of the medium, the cells were washed twice with PBS. Subsequently, 1 mL of 4% paraformaldehyde (Biosharp, China) was added to each well for fixation over a period of 15 minutes. This was followed by the addition of 1 mL of crystal violet ammonium oxalate solution (Solarbio, China) for 30 minutes at room temperature. The wells were then gently rinsed with double-distilled water. After allowing the cells to air dry, the cell clusters were photographed and counted. This experiment was conducted in triplicate.

### 2.9 Wound healing assay

Following trypsinization and cell counting, the cells were seeded into 6-well plates at a density of 50×10^4^ cells per well. Once the cells had reached confluence, scratches were made perpendicular to the initial horizontal line. Cell debris was subsequently removed using PBS, after administering mitomycin treatment to the cells for a duration of one hour, proceed to wash them with PBS, and fresh medium was added. The cells were then cultured at 37°C in a 5% CO_2_ atmosphere. The widths of the scratches were measured and recorded at 0h and 24h using an inverted biological microscope, with three fields of view captured at each time point.

### 2.10 Migration and invasion assay

Add 700 μL of medium (20% FBS, no P/S) to each well of a 24-well plate and subsequently place the chamber into the well containing the medium. Introduce 100 μL of the diluted cell suspension into the chamber, ensuring a total cell count of 10×10^4^. Incubate the cells under standard culture conditions for 24h (37°C, 5% CO_2_). After the incubation period, remove the chamber from the 24-well plate and gently eliminate any non-migrated cells and residual medium from the polycarbonate membrane using a cotton swab. Fix the cells with 4% paraformaldehyde for 30 minutes, followed by staining with 0.1% crystal violet for 15 minutes. Take a picture under the microscope and count the number of cells. The experiment was repeated 3 times.

### 2.11 Subcutaneous xenograft

NTG mice (4-6 weeks old) were purchased from Beijing Sibeifu Biotechnology Co., Ltd, and housed under special pathogen-free conditions. Animal experiments were conducted with the approval of the Animal Ethics Committee (NO: MDL2024-04-05-01), ensuring that all mice were provided with adequate water and food. Stable transfection of FTC133 and FTC238 cells with sh-PMAIP1 or sh-NC was conducted, followed by selection using puromycin. Subsequently, FTC133 and FTC238 cells with stable PMAIP1 knockdown were digested and resuspended in PBS at a concentration of 5×10^6^ cells per 100μL. These cells were then injected into the left axillary region of randomly assigned mice (5 mice per group) within one hour. The condition of the mice was monitored, and tumor volume measurements commenced on day 3. Tumor volume was assessed every 3 days using calipers and calculated using the formula: volume (mm³) = 0.5 × length × width². After the mice were euthanized, the tumors were subsequently isolated, photographed, and weighed. The excised tissues were either fixed in 10% neutral-buffered formalin or processed further. Tumor sections from paraffin-embedded blocks were utilized for histological examination.

### 2.12 Immunohistochemical staining

Following dewaxing and rehydration, the tissue sections were immersed in 0.01M citrate buffer and subjected to microwave heating to restore antigenicity. After incubation at room temperature for 10 minutes, the sections were treated with PBS and 1% periodate. Subsequently, the sections were incubated with the primary antibody overnight at 4°C, followed by a 30-minute incubation with the secondary antibody on the subsequent day. The sections were then subjected to a staining protocol that included diaminobenzidine staining, PBS washes, hematoxylin counterstaining, distilled water washes, and PBS rebluing. Dehydration was performed using a graded series of alcohols, with each stage lasting 5 minutes, and concluded with a 10-minute treatment in xylene. Finally, the sections were sealed with neutral gum for microscopic examination. Subsequently, three images of each tissue slice were randomly captured using a microscope, and the staining intensity of these images was analyzed utilizing Aipathwell software (Wuhan servicebio technology Co, Ltd). The staining intensity of the sections was evaluated based on the rate of positive cells and the positive area rate.

### 2.13 Transcriptome sequencing and analysis

Each experimental group comprised three samples, with each sample containing a volume of 1×10^7^ cells. Following RNA extraction, the integrity of the RNA was rigorously assessed using the Agilent 2100 Bioanalyzer. Upon completion of library construction, preliminary quantification was conducted using the Qubit 2.0 Fluorometer, and the libraries were subsequently diluted to a concentration of 1.5 ng/μL. Following the completion of quality checks, various libraries were pooled in accordance with the manufacturer’s guidelines, taking into account the necessary effective concentration and desired sequencing output. These pooled libraries were then subjected to Illumina sequencing. The resulting raw data underwent quality control and were aligned to the reference genome HG38 for annotation purposes. Comparative analysis of gene expression profiles was performed between sh-PMAIP1 cells and the control group. Subsequently, differentially expressed genes were identified, and enrichment analyses for Kyoto Encyclopedia of Genes and Genomes (KEGG) pathways were conducted utilizing the R (Version 3.5.0).

### 2.14 Statistical analysis

*In vitro* experiments were performed in triplicates. All statistical tests were performed using Graphpad Prism 8.0 (Graphpad Software Inc., San Diego, CA) or the SPSS program (Version 22.0; SPSS, Chicago, IL). The mean ± standard deviation was used for statistical description and the t-test for statistical analysis. Statistical significance was determined at *P* < 0.05. *P < 0.05, **P < 0.01, ***P < 0.001.

## 3 Results

### 3.1 PMAIP1 was upregulated in FTC

**Figure 1:**
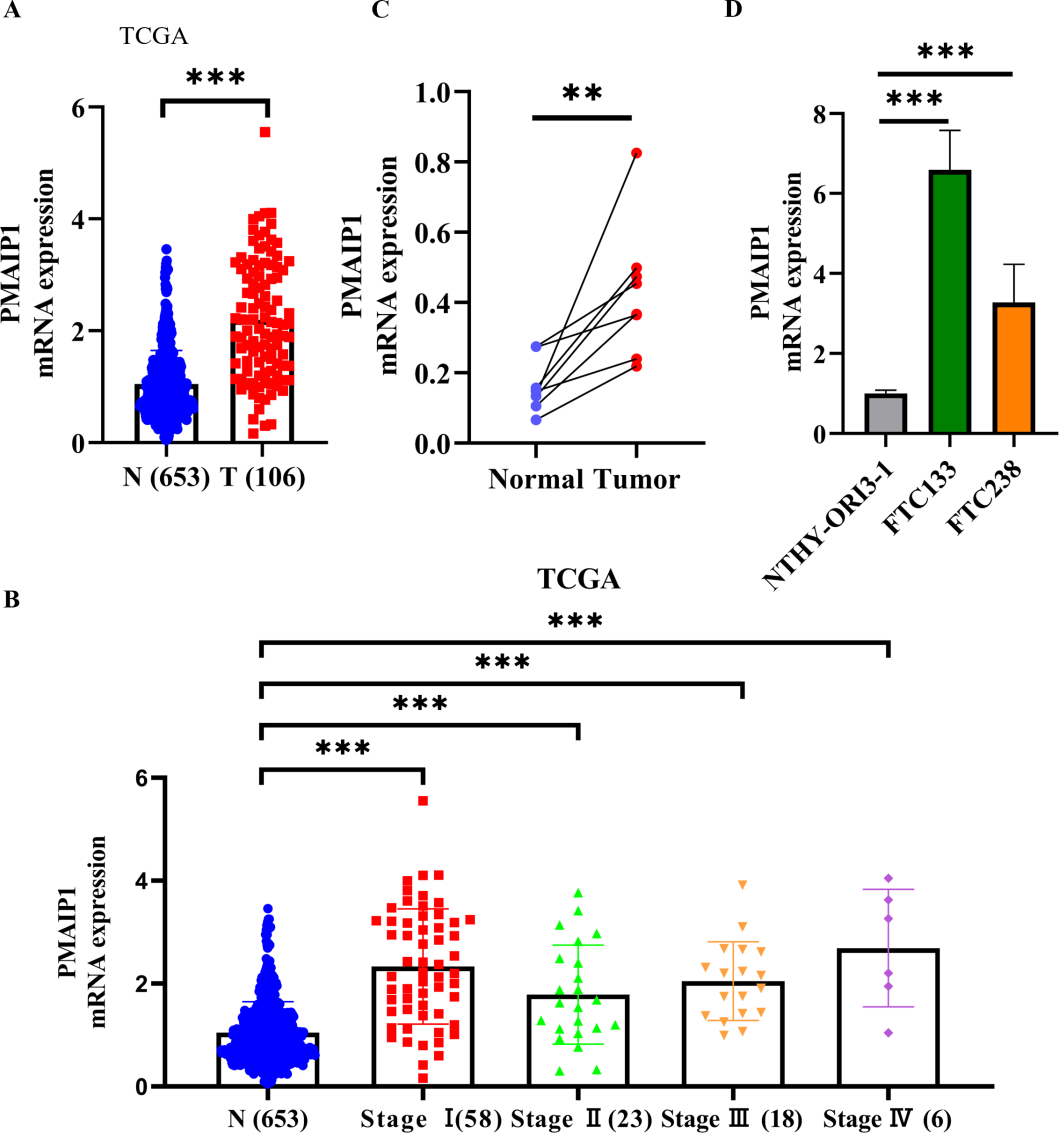
PMAIP1 was upregulated in FTC. **(A)** RNA-sequencing expression profiles and corresponding clinical data for FTC (n=106) were obtained from TCGA dataset. Normal control datasets (n=653) were sourced from the GTEx data portal. PMAIP1 mRNA expression was higher in FTC tissues relative to normal tissues at all stages, ***P < 0.001. **(B)** In stages I (n=58), II (n=23), III (n=18), and IV (n=6), the expression level of PMAIP1 was all significantly elevated in FTC tissues compared to normal tissues (n=653), ***P < 0.001. **(C)** 8 FTC tissues and 8 paired adjacent normal tissues were detected by qRT-PCR, showing that PMAIP1 expression was upregulated in FTC tissues compared with paired adjacent normal tissues, **P < 0.01. **(D)** Cell lines were used to explore the PMAIP1 level. Compared with NTHY-ORI3-1, PMAIP1 expression was upregulated in both FTC133 and FTC238, ***P < 0.001.

We acquired data on 106 FTC samples and 653 normal tissue samples from TCGA and GTEx databases. Comparative analysis of PMAIP1 expression between FTC and normal tissues revealed a significant overexpression of PMAIP1 in FTC tissues (**Figure 1A**). Additionally, we examined PMAIP1 expression across stages I-IV of FTC relative to normal tissues, finding elevated mRNA levels of PMAIP1 in FTC samples (**Figure 1B**). To further validate these findings, we collected 8 pairs of FTC and adjacent non-cancerous tissues and assessed PMAIP1 expression differences. The results demonstrated a significant upregulation of PMAIP1 expression in FTC tissues compared to adjacent non-cancerous tissues (**Figure 1C**). Subsequently, we assessed the expression levels of PMAIP1 across three cell lines. Among these, PMAIP1 was markedly overexpressed in the FTC cell lines FTC133 and FTC238 relative to NTHY-ORI3-1 (**Figure 1D**). These findings indicated that PMAIP1 was upregulated in FTC.

### 3.2 Knockdown of PMAIP1 significantly inhibited the proliferation and metastasis of FTC *in vitro*

**Figure 2:**
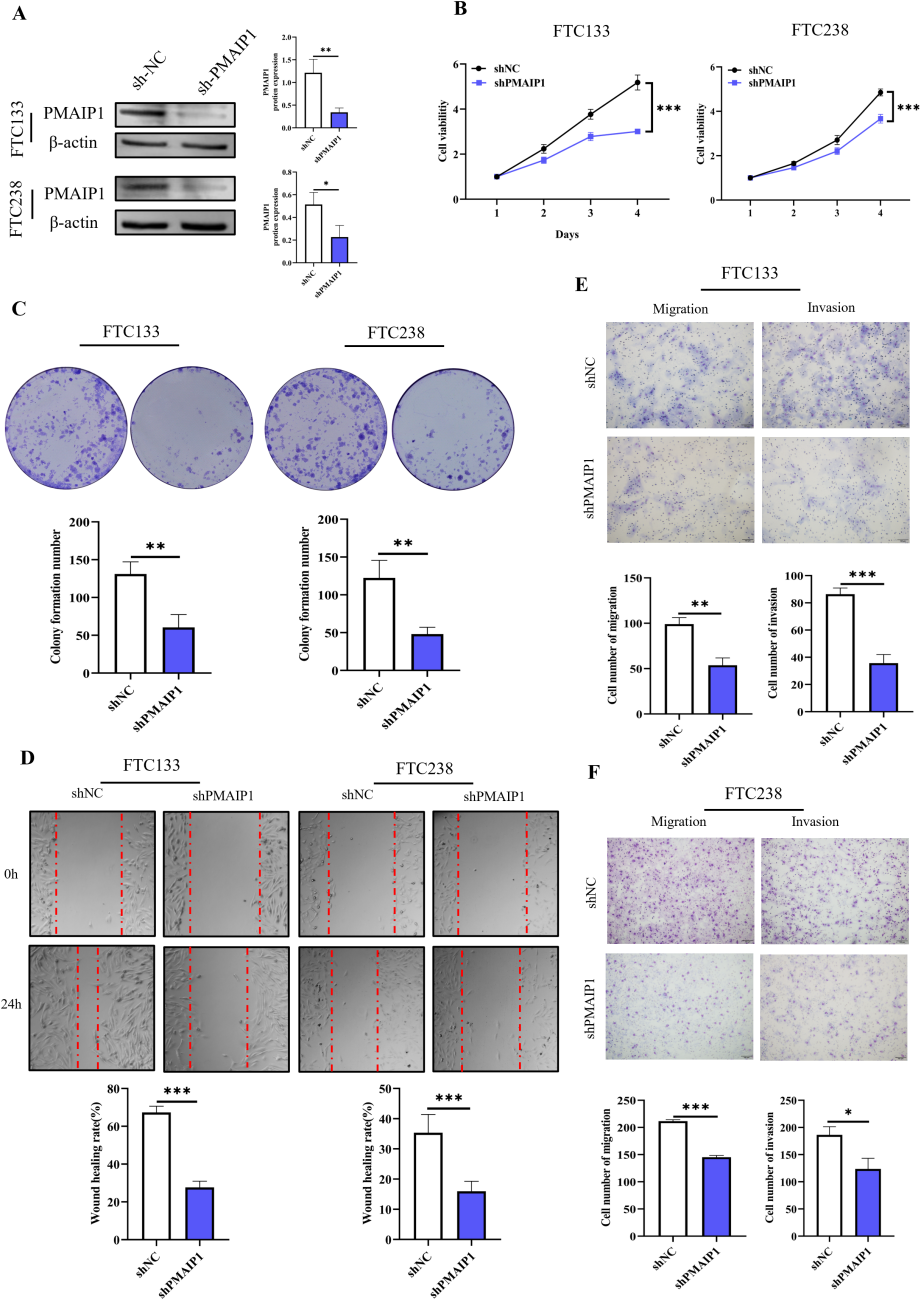
Knockdown of PMAIP1 significantly inhibited the proliferation and metastasis of FTC *in vitro*. **(A)** Stable knockdown of PMAIP1 by shRNA in FTC133 and FTC238 was confirmed by WB, *P < 0.05, **P < 0.01. **(B)** CCK-8 assay showed that the knockdown of PMAIP1 in FTC133 and FTC238 cells resulted in a significant decrease in cell proliferation compared with the NC group, ***P < 0.001. **(C)** Colony formation assay showed the knockdown of PMAIP1 markedly decreased the colony formation number of FTC133 and FTC238 cells compared with the NC group, **P < 0.01. **(D)** The wound healing assay suggested the knockdown of PMAIP1 substantially impaired the wound-healing ability of FTC133 and FTC238 cells, ***P < 0.001. **(E)** Migration and invasion assay showed the knockdown of PMAIP1 significantly reduced the number of invasive and migrating FTC133 cells, **P < 0.01, ***P < 0.001. **(F)** Migration and invasion assay showed the knockdown of PMAIP1 significantly decreased the number of invasive and migrating FTC238 cells, ***P < 0.001, *P < 0.05.

To construct cell lines with PMAIP1 knockdown, FTC133 and FTC238 cells were transfected with plasmids and subsequently screened using puromycin to construct cell lines with stable knockdown of PMAIP1 and detected by WB. The results indicated that PMAIP1 exhibits a high and stable knockdown efficiency in FTC133 and FTC238 cell lines (**Figure 2A**). CCK-8 assays demonstrated that the knockdown of PMAIP1 in these cell lines resulted in decreased cell proliferation (**Figure 2B**). Additionally, the knockdown of PMAIP1 significantly inhibited the colony formation ability of both FTC133 and FTC238 (**Figure 2C**). To further elucidate the impact of PMAIP1 on the migratory and invasive capabilities of FTC133 and FTC238 cells, wound healing and transwell assays were conducted. Quantitative analysis revealed that the wound healing rate was significantly reduced in the shPMAIP1 group compared to the shNC group (**Figure 2D**). Migration and invasion assays demonstrated that, in comparison to the shNC group, the knockdown of PMAIP1 significantly decreased the numbers of invasion and migration cells of FTC133 and FTC238 (**Figures 2E, F**).

### 3.3 Knockdown of PMAIP1 significantly inhibited the proliferation and metastasis of FTC *in vivo*

**Figure 3:**
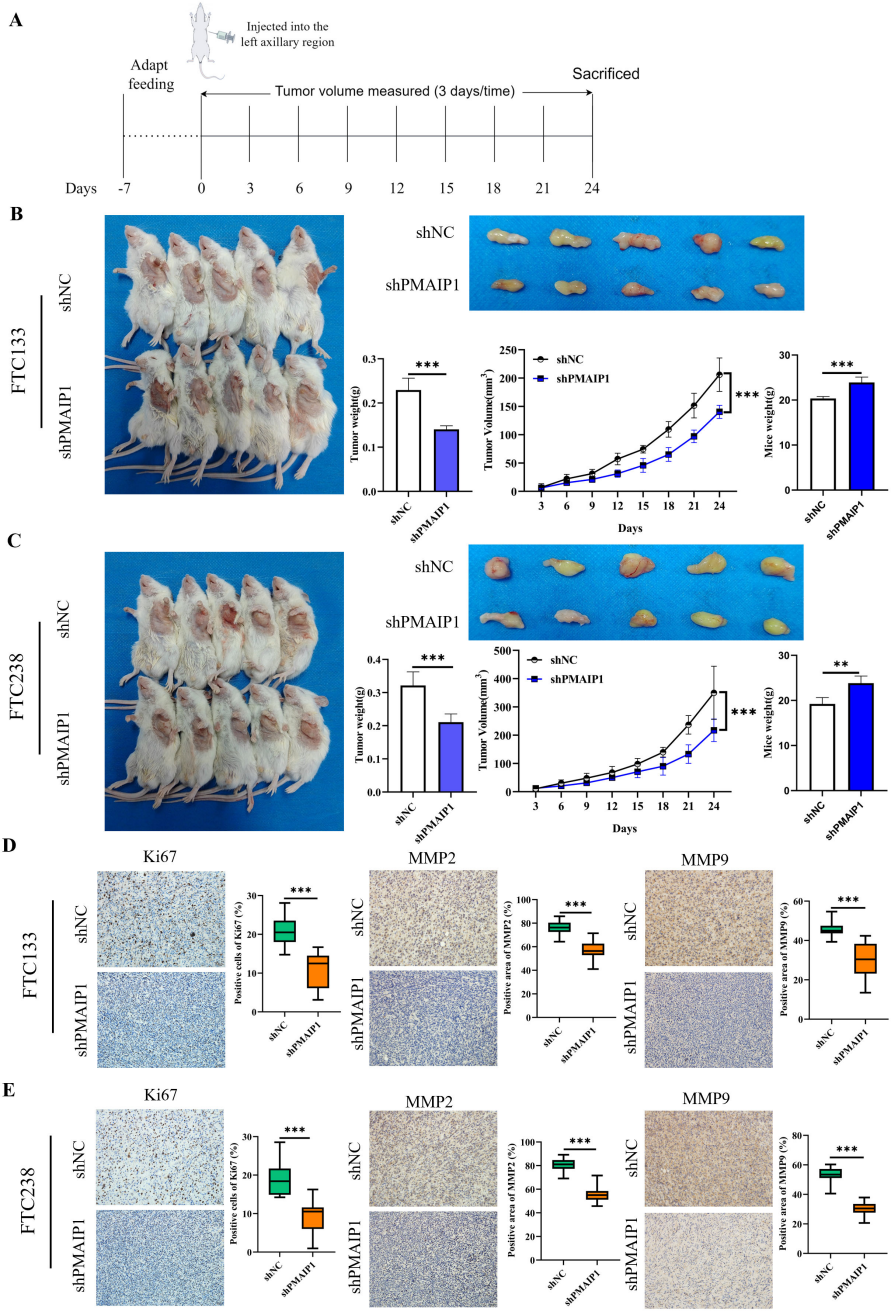
Knockdown of PMAIP1 significantly inhibited the proliferation and metastasis of FTC *in vivo*. **(A)** Experimental flowchart of subcutaneous tumor transplantation in NTG mice. **(B)** Compared with the NC group, the weight and volume of tumors of FTC133 were significantly reduced (***P < 0.001) and the body weight of mice was increased (***P < 0.001) by knockdown of PMAIP1. **(C)** Compared with the NC group, the weight and volume of tumors of FTC238 were significantly reduced (***P < 0.001) and the body weight of mice was increased (**P < 0.01) by knockdown of PMAIP1. **(D)** Knockdown of PMAIP1 decreased the Ki67 positive cells percentage, MMP2 and MMP9 positive area percentage in FTC133 subcutaneous xenografts determined by IHC staining, ***P < 0.001. **(E)** Knockdown of PMAIP1 decreased the Ki67 positive cells percentage, MMP2 and MMP9 positive area percentage in FTC238 subcutaneous xenografts determined by IHC staining, ***P < 0.001.

Initially, NTG mice underwent one week of adaptive feeding. Subsequently, FTC133 and FTC238 cells, with stable PMAIP1 knockdown and negative control, were subcutaneously injected into the left forelimb armpit of NTG mice, and tumor tissues were dissected post-experiment for further analysis (**Figure 3A**). The findings indicated that PMAIP1 knockdown significantly reduced tumor weight and tumor volume. In parallel, the mice in the shPMAIP1 group exhibited a significant increase in body weight compared to those in the shNC group (**Figures 3B, C**). To assess the impact of PMAIP1 knockdown on tumor proliferation and metastasis, we utilized the proliferation marker Ki67 and the metastasis markers MMP2 and MMP9 for further analysis. The expression levels of Ki67, MMP2, and MMP9 in tumor tissues were determined via immunohistochemical (IHC). The findings revealed that the expression levels of Ki67, MMP2, and MMP9 were reduced in the shPMAIP1 group compared with the shNC group (**Figures 3D, E**).

### 3.4 PMAIP1 activated the Wnt signaling pathway

**Figure 4:**
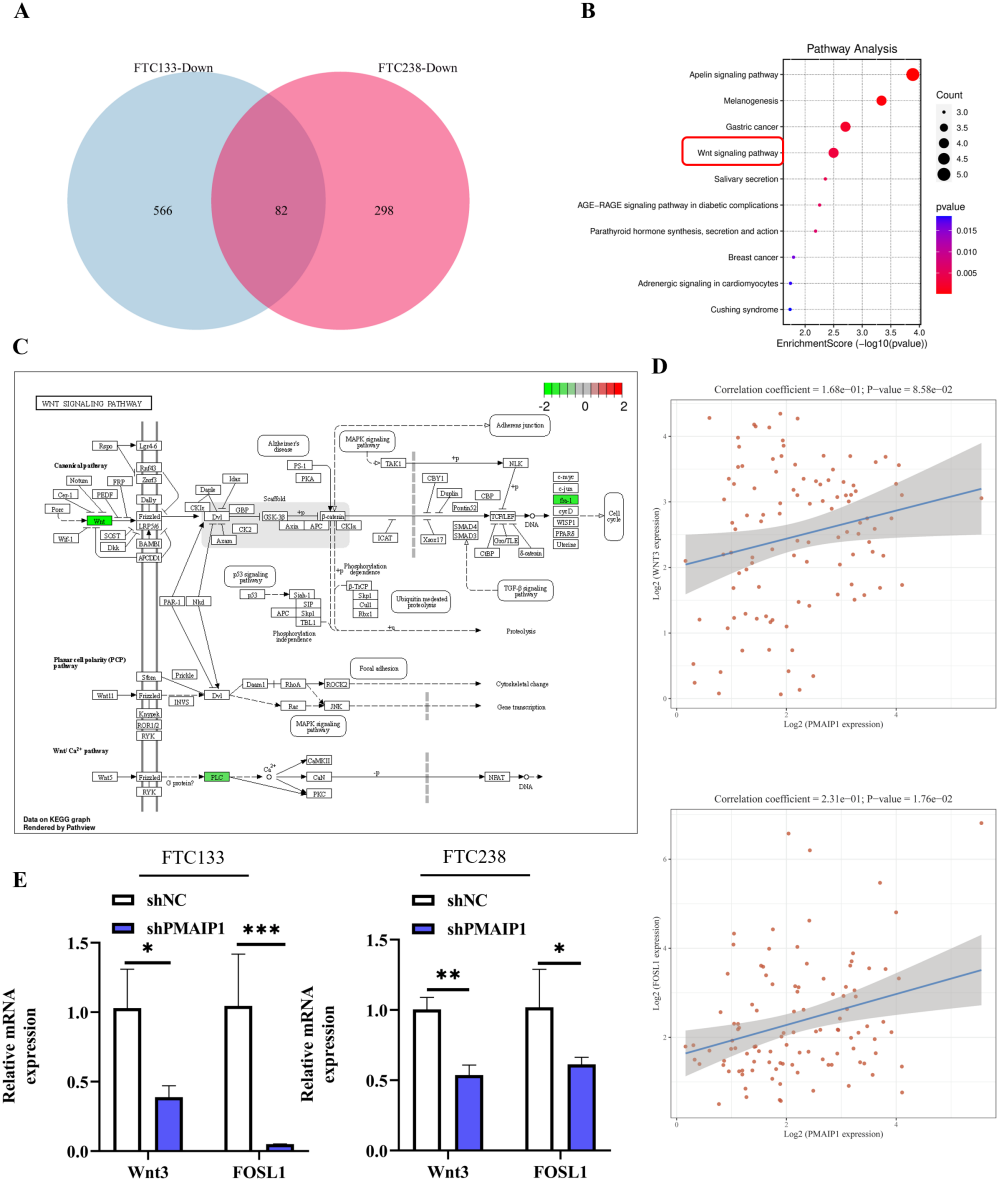
PMAIP1 activated the Wnt signaling pathway. **(A)** Venn diagram of downregulated genes in FTC133 and FTC238 cells and 82 common genes were detected. **(B)** The top 10 enrichment KEGG pathways of 82 intersection genes were shown. **(C)** As the Wnt signaling pathway showed, expression of Wnt, fra-1, and PLC were decreased after the knockdown of PMAIP1. **(D)** Spearman’s correlation analysis of TCGA dataset suggested that the expression level of PMAIP1 was directly proportional to Wnt3 in FTC and the expression levels of PMAIP1 are directly proportional to FOSL1 in FTC. **(E)** In FTC133 and FTC238 cell lines, the expression levels of Wnt3 and FOSL1 were both decreased after PMAIP1 was knocked down, *P < 0.05, **P < 0.01, ***P < 0.001.

We extracted RNA from cells of the shNC and shPMAIP1 groups in both FTC133 and FTC238 cell lines, followed by transcriptome sequencing. Differentially expressed genes were annotated to identify disease-associated genes. Intersection analysis of down-regulated genes from both cell lines revealed 82 genes (**Figure 4A**). Subsequent enrichment analysis of these 82 genes identified their association with several KEGG pathways, including the Apelin signaling pathway, Melanogenesis, Gastric cancer and the Wnt signaling pathway, et al. (**Figure 4B**). The schematic diagram of the Wnt signaling pathway shows that when PMAIP1 is knocked down, the expression levels of Wnt3, FOSL1, and PLC in the Wnt signaling pathway are correspondingly reduced. Interestingly, FOSL1 is a downstream gene of Wnt3. Therefore, we decided to explore the mechanism of the PMAIP1/Wnt3/FOSL1 pathway in FTC (**Figure 4C**). Additionally, we extracted data from 106 FTC patients from the TCGA database and conducted a correlation analysis, which revealed a direct proportional relationship between the expression levels of PMAIP1 and those of Wnt3 and FOSL1 (**Figure 4D**). These findings were consistent with the results obtained from the transcriptome sequencing analysis conducted in this study. Furthermore, we assessed the expression levels of Wnt3 and FOSL1 in both the shNC and shPMAIP1 groups and the results indicated that the knockdown of PMAIP1 led to a reduction in the expression levels of Wnt3 and FOSL1 (**Figure 4E**).

### 3.5 PMAIP1 modulated the proliferation and metastasis of FTC by regulating the Wnt signaling pathway

**Figure 5:**
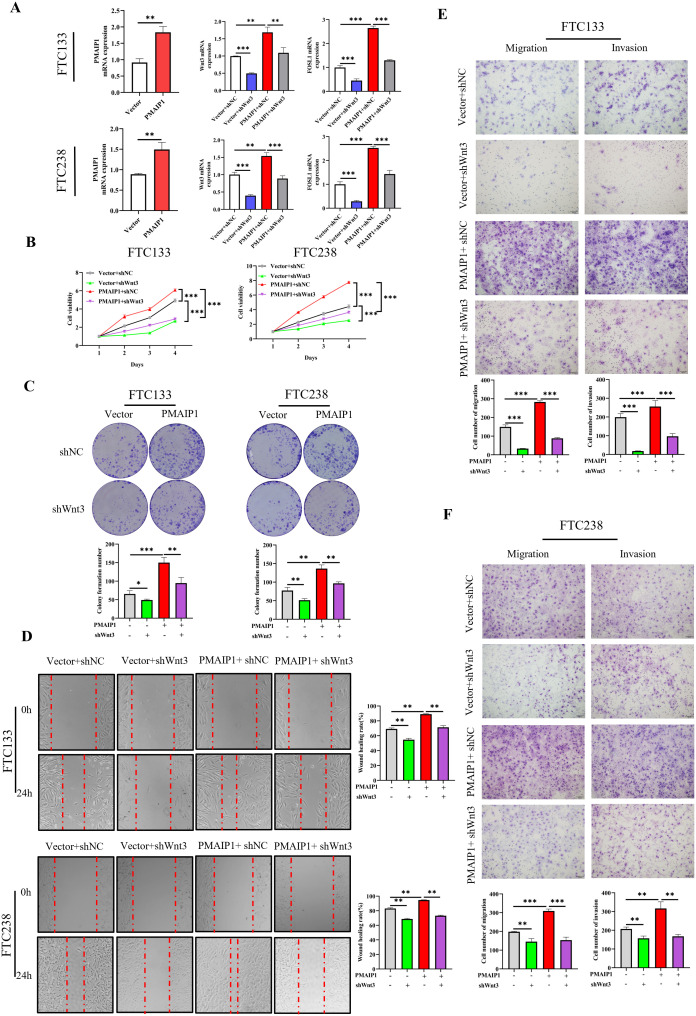
PMAIP1 modulated the proliferation and metastasis of FTC by regulating the Wnt signaling pathway. **(A)** Overexpression of PMAIP1 in FTC133 and FTC238 was confirmed by qRT-PCR. The overexpression of PMAIP1 led to elevated levels of Wnt3 and FOSL1. Conversely, the expression levels of Wnt3 and FOSL1 were significantly diminished in the Vector+shWnt3 group compared to the Vector+shNC group; in the meantime, a comparative analysis revealed that the expression levels of Wnt3 and FOSL1 were significantly reduced in the PMAIP1+shWnt3 group relative to the PMAIP1+shNC group, **P < 0.01, ***P < 0.001. **(B)** CCK-8 assay was used to determine the proliferation ability of cells. The proliferation ability of FTC133 and FTC238 in the PMAIP1+shNC group were promoted compared with the Vector+shNC group, ***P < 0.001. Compared with the Vector+shNC group, the knockdown of Wnt3 significantly decreased the proliferation ability of FTC133 and FTC238 in the Vector+shWnt3 group, ***P < 0.001. Compared with the PMAIP1+shNC group, the knockdown of Wnt3 significantly decreased the proliferation ability of FTC133 and FTC238 in the PMAIP1+shWnt3 group, ***P < 0.001. **(C)** The colony formation numbers of FTC133 and FTC238 in the PMAIP1+shNC group were promoted compared with the Vector+shNC group, **P < 0.01, ***P < 0.001. Compared with the Vector+shNC group, knockdown of Wnt3 significantly decreased the colony formation numbers of FTC133 and FTC238 in the Vector+shWnt3 group, *P < 0.05, **P < 0.01. Compared with the PMAIP1+shNC group, knockdown of Wnt3 significantly decreased the colony formation numbers of FTC133 and FTC238 in the PMAIP1+shWnt3 group, **P < 0.01. **(D)** The wound-healing rates of FTC133 and FTC238 in the PMAIP1+shNC group were promoted compared with the Vector+shNC group, **P < 0.01. Compared with the Vector+shNC group, the knockdown of Wnt3 significantly decreased the wound-healing rate of FTC133 and FTC238 in the Vector+shWnt3 group, **P < 0.01. Compared with the PMAIP1+shNC group, the knockdown of Wnt3 significantly decreased the wound-healing rate of FTC133 and FTC238 in the PMAIP1+shWnt3 group, **P < 0.01. **(E, F)** Migration and invasion cell numbers of FTC133 and FTC238 in the PMAIP1+shNC group were increased compared with the Vector+shNC group, **P < 0.01, ***P < 0.001. Compared with the Vector+shNC group, migration and invasion cells of FTC133 and FTC238 significantly decreased in the Vector+shWnt3 group, **P < 0.01, ***P < 0.001. Compared with the PMAIP1+shNC group, migration and invasion cells of FTC133 and FTC238 significantly decreased in the PMAIP1+shWnt3 group, **P < 0.01, ***P < 0.001.

To elucidated the role of PMAIP1 in FTC cancer progression via the Wnt pathway, we conducted a rescue experiment employing shWnt3. Initially, we engineered FTC133 and FTC238 cell lines to overexpress PMAIP1 (**Figure 5A**). The results indicated that the overexpression of PMAIP1 led to elevated levels of Wnt3 and FOSL1. Conversely, the expression levels of Wnt3 and FOSL1 were significantly diminished in the Vector+shWnt3 group compared to the Vector+shNC group; in the meantime, a comparative analysis revealed that the expression levels of Wnt3 and FOSL1 were significantly reduced in the PMAIP1+shWnt3 group relative to the PMAIP1+shNC group (**Figure 5A**). This indicated that the knockdown of Wnt3 was successful. The CCK-8 assays demonstrated that cell proliferation in the PMAIP1+shNC group was increased compared with the Vector+shNC group. Furthermore, compared with the Vector+shNC group, proliferation of cells of the Vector+shWnt3 group was significantly decreased. Compared with the PMAIP1+shNC group, proliferation of cells of the PMAIP1+shWnt3 group was significantly decreased (**Figure 5B**). Cell colony formation numbers of the PMAIP1+shNC group was increased compared with the Vector+shNC group. Compared with the Vector+shNC group, cell colony formation numbers of the Vector+shWnt3 group was significantly decreased. Compared to the PMAIP1+shNC group, cell colony formation numbers of the PMAIP1+shWnt3 group was significantly decreased (**Figure 5C**). The wound healing assays demonstrated that the wound healing rate of the PMAIP1+shNC group was increased compared with the Vector+shNC group. Furthermore, compared with the Vector+shNC group, the wound healing rate of the Vector+shWnt3 group was significantly decreased. Compared to the PMAIP1+shNC group, the wound healing rate of the PMAIP1+shWnt3 group was significantly decreased (**Figure 5D**). Migration and invasion assays revealed that the cell invasion and migration numbers of the PMAIP1+shNC group was increased compared with the Vector+shNC group. Compared with the Vector+shNC group, cell numbers of the Vector+shWnt3 group was significantly decreased. Compared to the PMAIP1+shNC group, cell numbers of the PMAIP1+shWnt3 group was significantly decreased (**Figures 5E, F**).

### 3.6 PMAIP1 regulated the proliferation and metastasis of FTC by FOSL1

**Figure 6:**
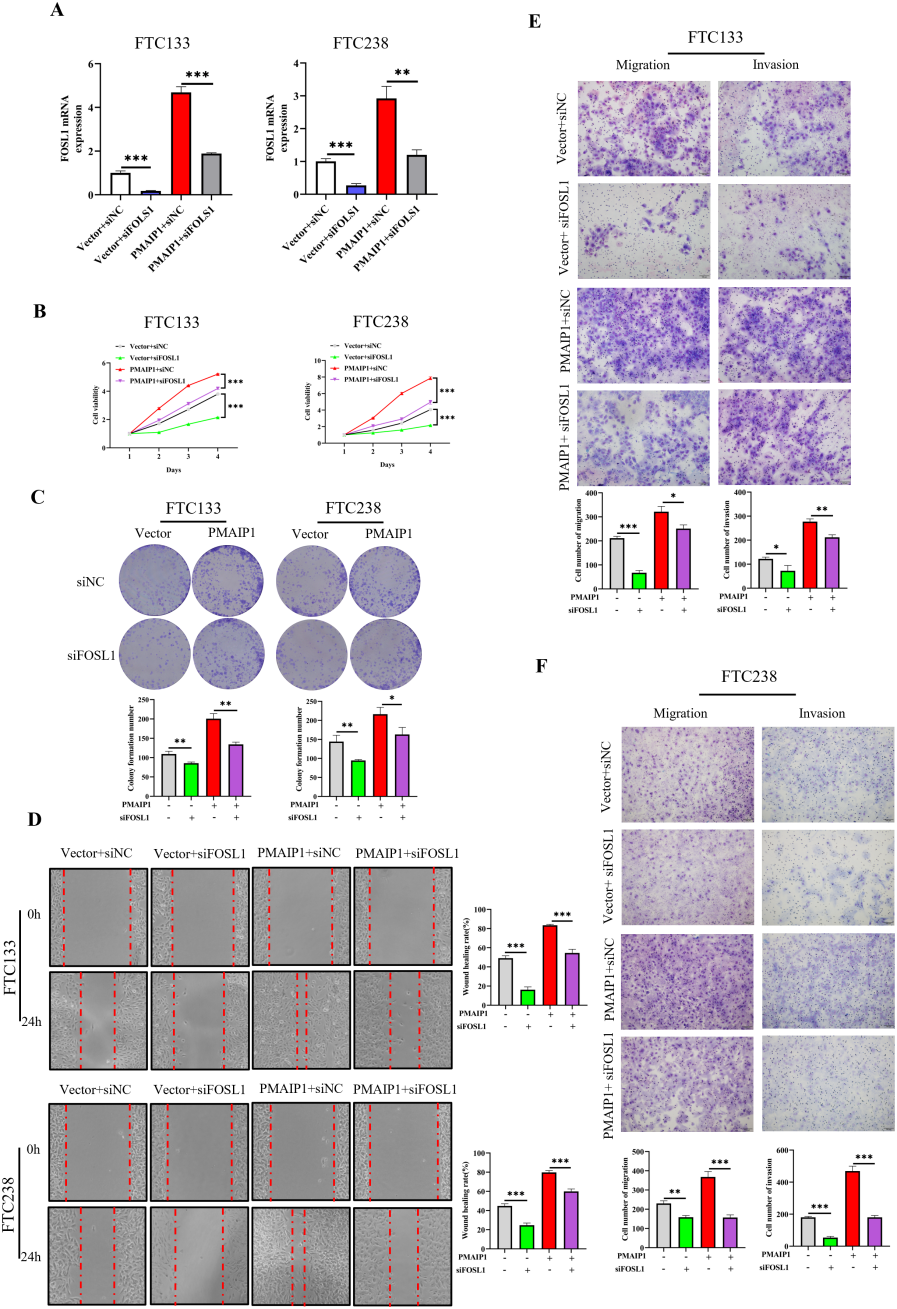
PMAIP1 regulated the proliferation and metastasis of FTC by FOSL1. **(A)** Knockdown of FOSL1 in FTC133 and FTC238 was confirmed by qRT-PCR. The expression level of FOSL1 were significantly diminished in the Vector+siFOSL1 group compared to the Vector+siNC group; the expression level of FOSL1 were significantly reduced in the PMAIP1+ siFOSL1 group relative to the PMAIP1+siNC group, **P < 0.01, ***P < 0.001. **(B)** Compared with the Vector+siNC group, the knockdown of FOSL1 significantly decreased the proliferation ability of FTC133 and FTC238 in the Vector+siFOSL1 group, ***P < 0.001. Compared with the PMAIP1+siNC group, the knockdown of FOSL1 significantly decreased the proliferation ability of FTC133 and FTC238 in the PMAIP1+siFOSL1 group, ***P < 0.001. **(C)** Compared with the Vector+siNC group, the knockdown of FOSL1 significantly decreased the colony formation numbers of FTC133 and FTC238 in the Vector+siFOSL1 group, *P < 0.05, **P < 0.01. Compared with the PMAIP1+siNC group, knockdown of FOSL1 significantly decreased the colony formation numbers of FTC133 and FTC238 in the PMAIP1+siFOSL1 group, **P < 0.01. **(D)** Compared with the Vector+siNC group, knockdown of FOSL1 significantly decreased the wound-healing rate of FTC133 and FTC238 in the Vector+siFOSL1 group, ***P < 0.001. Compared with the PMAIP1+siNC group, knockdown of FOSL1 significantly decreased the wound-healing rate of FTC133 and FTC238 in the PMAIP1+siFOSL1 group, ***P < 0.001. **(E, F)** Compared with the Vector+ siNC group, migration and invasion cells of FTC133 and FTC238 significantly decreased in the Vector+siFOSL1 group, *P < 0.05, **P < 0.01, ***P < 0.001. Compared with the PMAIP1+siNC group, migration and invasion cells of FTC133 and FTC238 significantly decreased in the PMAIP1+ siFOSL1 group, *P < 0.05, **P < 0.01, ***P < 0.001.

These experimental results suggested that PMAIP1 influences the cancer progression of FTC through the Wnt pathway. However, the potential involvement of FOSL1 in this process remains to be elucidated. To investigate this, we transfected FTC133 and FTC238 cells with siRNA targeting FOSL1. The results indicated that, in comparison to the Vector+siNC group, the expression level of FOSL1 was significantly reduced in the Vector+siFOSL1 group: in comparison to the PMAIP1+siNC group, the expression level of FOSL1 in the PMAIP1+ siFOSL1 group was significantly reduced, indicating successful siRNA transfection (**Figure 6A**). The CCK-8 assays demonstrated that, compared with the Vector+siNC group, proliferation of cells of the Vector+siFOSL1 group was significantly decreased. Compared with the PMAIP1+siNC group, proliferation of cells of the PMAIP1+ siFOSL1 group was significantly decreased (**Figure 6B**). Compared with the Vector+siNC group, cell colony formation numbers of the Vector+siFOSL1 group was significantly decreased. Compared to the PMAIP1+siNC group, cell colony formation numbers of the PMAIP1+siFOSL1 group was significantly decreased (**Figure 6C**). The wound healing assays demonstrated that, compared with the Vector+siNC group, the wound healing rate of the Vector+siFOSL1 group was significantly decreased. Compared to the PMAIP1+siNC group, the wound healing rate of the PMAIP1+siFOSL1 group was significantly decreased (**Figure 6D**). Migration and invasion assays revealed that, compared with the Vector+siNC group, cell numbers of the Vector+siFOSL1 group was significantly decreased. Compared to the PMAIP1+siNC group, cell numbers of the PMAIP1+siFOSL1 group was significantly decreased (**Figures 6E, F**).

## 4 Discussion

Our study innovatively confirmed that PMAIP1 could impact the progression of FTC by regulating the Wnt3/FOSL1 pathway, thereby offered new therapeutic possibilities for FTC. FTC is the second most common histotype of differentiated thyroid cancer, often overshadowed by the more prevalent PTC (13). However, the propensity of FTC for distant metastasis and poor prognosis renders it a critical subtype of thyroid cancer that warrants significant attention (5). Despite the growing body of research on FTC over the years, its pathogenesis remains largely elusive. Identifying potential biomarkers is crucial for advancing our understanding of FTC pathogenesis and for identifying potential therapeutic targets. In this study, we analyzed data from FTC and normal tissues available in the TCGA and GETx databases. Our findings revealed that the expression level of PMAIP1 was significantly elevated in FTC tissues compared to normal tissues. Notably, PMAIP1 was also overexpressed in FTC stages I-IV, suggested that its overexpression may influence the progression of FTC. Furthermore, validation using collected FTC tissues and FTC cell lines yielded results consistent with our initial analysis. Consequently, we hypothesized that PMAIP1 is overexpressed in FTC and may exert a pro-cancer effect.

To validate our hypothesis, we achieved stable knockdown of PMAIP1 in two cell lines, FTC133 and FTC238, resulting in a marked inhibition of cell proliferation and metastatic capability. Additionally, we established a subcutaneous xenograft model using immunodeficient mice. The findings demonstrated that PMAIP1 knockdown significantly suppressed the growth of FTC tumors *in vivo*, as evidenced by reductions in both tumor weight and volume. This study demonstrated that the knockdown of PMAIP1 inhibited the progression of FTC both *in vivo* and *in vitro*. Ki67 was utilized as a significant marker for cell proliferation, while MMP2 and MMP9 served as critical markers for distant metastasis (14–16). IHC results revealed that the knockdown of PMAIP1 was associated with reduced expression levels of Ki67, MMP2, and MMP9. These findings suggested that targeting PMAIP1 could effectively inhibit FTC growth and metastasis, thereby expanding clinical treatment options for FTC.

Additionally, PMAIP1 has been implicated in various biological processes. For instance, long non-coding RNA LOC101928963 regulated the proliferation and apoptosis of spinal glioma by interacting with PMAIP1, providing a foundation for targeted therapy of spinal glioma (17). Plant Homeo Domain Finger Protein 8 regulated mesodermal and cardiac differentiation of embryonic stem cells by mediating the histone demethylation of PMAIP1 (18). Another study elucidated a novel non-epigenetic mechanism of action for the hypomethylating agent 5-azacitidine and its synergistic activity with the BCL-2 selective inhibitor venetoclax. This combination, through the ISR-mediated induction of PMAIP1, could reduce drug resistance in acute myeloid leukemia (19). These findings suggested that PMAIP1 was a critical regulator in disease development and treatment. However, there are limited studies on the role of PMAIP1 in TC. Bortezomib sensitized TC cells to Vemurafenib through mitochondrial dysregulation and the induction of apoptosis, which was accompanied by an increased expression of NOXA/PMAIP1 (20). In this study, the knockdown of PMAIP1 significantly inhibited the proliferation and metastasis of FTC. To elucidate the pro-cancer role of PMAIP1 in FTC, we conducted further investigations. Transcriptome sequencing was utilized to identify differentially expressed genes associated with PMAIP1. Enrichment analysis of the down-regulated gene set revealed an inhibition of the Wnt signaling pathway, accompanied by the down-regulation of Wnt3 and FOSL1. Bioinformatics analysis revealed a direct proportionality between PMAIP1 and the expression levels of Wnt3 and FOSL1, corroborating the findings from transcriptome sequencing. Additionally, we verified that the expression patterns of Wnt3 and FOSL1 were consistent with those of PMAIP1. Overexpression of PMAIP1 resulted in increased expression levels of Wnt3 and FOSL1 as well. Simultaneously, we conducted rescue experiments and verified that the concurrent knockdown of Wnt3, in the context of PMAIP1 overexpression, significantly inhibited the proliferation and metastasis of FTC. These findings suggesteded that PMAIP1 may influence the proliferation and metastasis of FTC through modulation of the Wnt signaling pathway. It is well established that the Wnt signaling pathway was among the most frequently dysregulated pathways in human malignancies, playing pivotal roles in tumorigenesis and targeted therapy (21, 22). Currently, the functional mechanisms of the Wnt signaling pathway have been reported in breast cancer (23), PTC (24), prostate cancer (25) and gastric cancer (26). Notably, the relationship between PMAIP1 and the Wnt signaling pathway has not been previously documented in the literature. Our study may be the first to elucidated the regulation of FTC by PMAIP1 via the Wnt signaling pathway. Consequently, the findings of this research offered a more practical and feasible approach to the clinical management of FTC.

Current research on the apelin pathway primarily centered on its implications for cardiovascular and metabolic disorders. Apelin, a peptide ubiquitously expressed throughout the body, engaged the apelin receptor to facilitate endothelium-dependent vasodilation and inotropy, lower blood pressure, and promote angiogenesis. The apelin system was posited to confer protection against arrhythmias, inhibit thrombosis, and exert significant anti-fibrotic effects (27). Additionally, empirical studies have demonstrated that apelin influences glucose and lipid metabolism and modulates insulin secretion (28). Moreover, evidence suggested that the apelin system was involved in inflammatory responses (29). On the other hand, components of the Wnt signaling pathway have been established as reliable biomarkers and potential targets for cancer therapy. Ongoing or completed clinical trials involving Wnt signaling pathway components indicated that Wnt-targeted therapies hold promising applications in clinical settings (30). Furthermore, updates on inhibitors, antagonists, and activators of the Wnt signaling pathway presented innovative strategies for personalized cancer treatment (21). This emphasized our focus on the Wnt signaling pathway. Our discovery found that PMAIP1 played a cancer-promoting role in FTC, representing a significant finding and identifying a potential target for FTC treatment. This rationale was compelling. However, we aimed for our research to inform clinical practices in the treatment of FTC and to facilitate the rapid translation of these findings into patient benefits. Therefore, investigating the regulatory relationship between the novel FTC oncogene PMAIP1 and the Wnt pathway during FTC progression opened new avenues for dual-targeted therapy of this malignancy, which holds substantial clinical significance. While we prioritized our investigation into the Wnt pathway, we did not overlook Apelin’s potential role in FTC. Moving forward, we will continue to monitor and explore various signaling pathways in FTC—including the Apelin signaling pathway.

Although we have demonstrated that PMAIP1 exerts its effects on FTC through the Wnt signaling pathway. Furthermore, modulation of PMAIP1 expression, either through knockdown or overexpression, correspondingly resulted in the down-regulation or up-regulation of FOSL1 expression levels. FOSL1 (also known as FRA-1) functions downstream of the Wnt signaling pathway and was implicated in promoting metastasis in head and neck squamous cell carcinoma, suggested its role as an oncogene (31). This raised the question of whether PMAIP1 exerts a pro-cancer effect on FTC by regulating FOSL1 downstream of Wnt. To address this, a series of rescue experiments were conducted in this study. The results demonstrated that the proliferation and metastasis of FTC cells were significantly inhibited following the knockdown of FOSL1. The same results were observed when FOSL1 was knocked down in the FTC cell line stably overexpressing PMAIP1. Consequently, we posited that our study substantiates, at least in part, the role of PMAIP1 in regulating the cancer progression of FTC via modulation of the Wnt3/FOSL1 pathway. FOSL1, a transcription factor, has been shown in previous studies to be highly upregulated in bile duct cancer in both humans and mice, correlating with poorer survival outcomes (32). Additionally, FOSL1 has been demonstrated to promote the proliferation, invasion, migration, and epithelial-mesenchymal transition of colorectal cancer cells, and was significantly associated with poor prognosis (33). These findings align with our research outcomes. Our results indicated that FOSL1 contributes to the oncogenesis of FTC by enhancing its proliferation and metastasis, a process regulated by PMAIP1 through the Wnt signaling pathway. This mechanism, which we have identified, could be critical for the progression of FTC and holds potential clinical significance.

Our study has several limitations. Firstly, the sample size of FTC tissues was limited, attributable to the difficulties associated with the clinical diagnosis of FTC. Secondly, the precise interaction between PMAIP1 and the Wnt signaling pathway remains unclear. In future studies, we will continue to collect FTC samples to further substantiate our findings. Concurrently, we will delve deeper into the mechanisms of action of PMAIP1.

In conclusion, our research demonstrated that PMAIP1 was overexpressed in FTC and correlated with the clinical stage of the disease. PMAIP1 emerged as a novel pro-cancer factor in FTC, and its knockdown significantly inhibited the proliferation and metastasis of FTC both *in vivo* and *in vitro*. Mechanistically, PMAIP1 regulated FOSL1 by modulating the Wnt signaling pathway, thereby promoting FTC progression. Targeting PMAIP1 may present a promising therapeutic strategy for FTC.

## Data Availability

The RNA data presented in this article has been deposited to the NCBI Repository, under accession numbers PRJNA1212579.
